# Outcomes of removing the fracture fragments in the treatment of intracapsular condylar fractures in children

**DOI:** 10.4317/medoral.27079

**Published:** 2025-02-15

**Authors:** Jia-Wei Dai, Chao Gong, Drissa Diarra, Zhi Li

**Affiliations:** 1 State Key Laboratory of Oral and Maxillofacial Reconstruction and Regeneration; Key Laboratory of Oral Biomedicine Ministry of Education; Hubei Key Laboratory of Stomatology; School and Hospital of Stomatology, Wuhan University, China; 2 Department of Oral and Maxillofacial Surgery, School and Hospital of Stomatology, Wuhan University, China

## Abstract

**Background:**

Treatment methods for mandibular condylar fractures in children can be broadly divided into closed treatment and open treatment (open reduction and internal fixation). The aim of the study is to evaluate the feasibility of removing the fracture fragments when treating intracapsular condylar fractures in children.

**Material and Methods:**

A retrospective study was performed in patients aged ≤12 years with intracapsular condylar fractures treated with removal of the fracture fragments from June 2010 through June 2018. The preoperative and postoperative data of physical complaints, facial asymmetry, maximal interincisal distance, occlusal relationship and radiographic examinations were extracted from the patients’ records. The collected preoperative and postoperative data were analysed.

**Results:**

Thirteen intracapsular condylar fractures in nine cases were subjected to fracture fragments removal. In these patients, clinical and radiographic results at different follow-up periods displayed normal occlusion and satisfactory bone healing. New condyles were found to be regenerated, in the cases with follow-up period longer than 3 months.

**Conclusions:**

Removal of fracture fragments proves to be effective in delivering satisfactory clinical results and permitting ongoing condyle remodelling and regeneration.

** Key words:**Condylar fractures, surgical management, condyle regeneration, child.

## Introduction

Condylar fracture is reportedly the most common type of mandibular fracture in children (1). It is suggested that, if not appropriately addressed, a paediatric condylar fracture may cause concern for abnormal growth or complications such as facial growth disturbances and temporomandibular joint (TMJ) disorders (2,3). No consensus 'gold standard' treatment exists for condylar fractures, and there is continued debate on whether condylar fractures should undergo surgical or conservative management (4). Surgical treatment, defined as open reduction and internal fixation, is uncommonly applied in paediatric condylar fractures (5,6). A recommended conservative management is the restoration of normal occlusion, with a short period of maxillomandibular fixation (MMF), followed by early physical therapy (7). Most surgeons choose conservative management as the first choice for satisfactory clinical outcomes in children (8,9).

Although closed treatment often leads to satisfactory outcomes in children, complications such as mandibular maldevelopment, TMJ dysfunction, and ankylosis can occur in patients who have had closed treatment (10). Most studies on closed treatment have been performed in patients with fewer displaced fractures and milder clinical symptoms (11,12). Reports on open treatment still need to be improved, and evidence has yet to show open treatment to be inferior. As observed in our clinical work, under any circumstances, intracapsular condylar fractures with comminution or severe dislocation, as well as restricted mouth opening, may result in TMJ ankylosis. On this basis, our working hypothesis was that the incidence of TMJ ankylosis would significantly decrease if the fracture fragments were removed to reduce the interference in mandibular movement. The present study describes this method and presents the clinical and radiographic outcomes of nine paediatric patients treated with it, aiming to evaluate its feasibility when treating intracapsular condylar fractures in children.

## Material and Methods

This retrospective study included all paediatric patients aged ≤12 years with intracapsular condylar fractures, presented to the authors' department and treated with removal of the fracture fragments from June 2010 through June 2018 and followed up for >3 months. The criteria for using this method were as follows:

1. Comminution of the condylar head or severe dislocation of the condylar fracture fragments out of the fossa.

2. Fragments interfere with mandibular movement, and the maximal mouth opening was less than one finger width (the patient's finger).

3. Persistent joint pain during mouth opening.

The patient's records contained preoperative data on physical complaints, facial asymmetry, maximal interincisal distance, and occlusal relationship. Preoperative spiral multislice computed tomography (CT) images were obtained to ascertain the location of the fracture, the degree of fragment displacement, and its severity.

The operation was performed under general anaesthesia. An incision was made over the preauricular area to access the fractured condyle, and the condyle was exposed after opening the capsule. The fracture fragments, blood clots and fibrous callus tissues were removed carefully, and the cartilage on the top of the condylar stump was maintained. Dissection of the displaced disc from the surrounding tissue was carefully performed, and then the disc was cautiously sutured if it presented torn or perforated. After that, the disc was repositioned over the top of the condylar stump, the lateral aspect of the disc was sutured to the soft tissue of the zygomatic root and its original temporal attachments, which were undamaged simultaneously, were retained. The mouth opening was checked during the operation. A soft diet was required for all patients for the first month after the surgery. The mouth-opening exercises were instructed early postoperatively and continued during the follow-up period.

Postoperative data on physical complaints, facial asymmetry, maximal interincisal distance, and occlusal relationships were collected from each follow-up. Simultaneously, spiral multislice CT of one week, three months and six months after surgery were obtained and analysed statistically.

**Figure 1:**
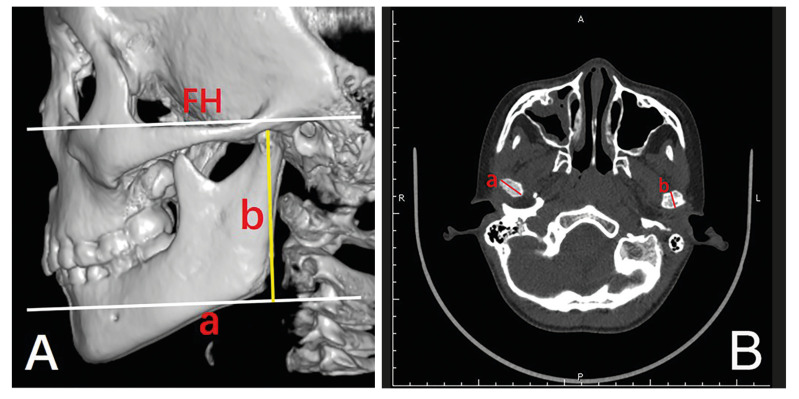
A) The measurement methods of MRH in the spiral multi-slice CT image: a line parallel to the Frankfort horizontal (FH) plane through the point of the ante gonial notch; b, ramus height: distance from the most superior end of the condyle to line a; B) The measurement methods of APD and MLD of the condylar process based on axial plane image: a mediolateral diameter of the condylar process; b, anteroposterior diameter of the condylar process).

Postoperative clinical evaluation included 1) occlusion, 2) maximal interincisal distance, 3) pain in mandibular movement and 4) functional or growth disturbance in the mandible. Radiographically evaluated parameters included 1) radiographic imaging appearance of the condyle, 2) mandibular ramus height (MRH), 3) anteroposterior diameter (APD) and 4) mediolateral diameter (MLD) of the condylar head (Fig. 1). The MRH was measured in the spiral multislice CT image, according to Chang *et al*. (13).

All values, presented as mean ± standard deviation, were obtained separately for the left and right sides, both postoperatively and at long-term follow-up. The students' paired t-test was conducted using IBM SPSS version 24.0. Statistical significance was defined at *p* < 0.05.

## Results

Nine paediatric patients with intracapsular condylar fractures treated with removal of fracture fragments at the authors’ department were included in the study, out of which seven were males (78%) and two were females (22%) with an average age of 9.2 years (range = 5-12 years). Three patients sustained unilateral condylar fractures, and six had bilateral fractures, among which two cases received removal of the fragments on only one side. In contrast, the other side was managed conservatively. Five patients had mandibular fractures: three were in the symphysis region, and two were in the body. All nine patients were followed up for 3 to 45 months (mean = 13.6 months). Among these patients, three follow-up periods were longer than 1 year.

This study included nine patients with 13 intracapsular condylar fractures treated with the removal of fracture fragments. Preoperative radiographic examination showed comminuted fractures in three condyles and severe dislocation of the condylar fracture fragments in ten condyles. Preoperative clinical examination revealed restriction of mouth opening and pains during mandibular movement in all patients (Table 1).

Surgical wound healing was achieved uneventfully in all cases. The maximal interincisal distance was more than three-finger width in all patients at 3 months after the operation (Table 1). The results showed that all nine patients displayed alignment and appropriate occlusion without any discrepancy, evident improvement in mouth opening and masticatory function restored without pain complaints at the last follow-up. Moreover, no instance of facial nerve injuries or TMJ ankylosis was found during the follow-up. In the five cases treated surgically on the unilateral condyle, we found no growth disturbance on the treated condyles compared with the opposite side at 6 months and 2 years after surgery.

**Figure 2:**
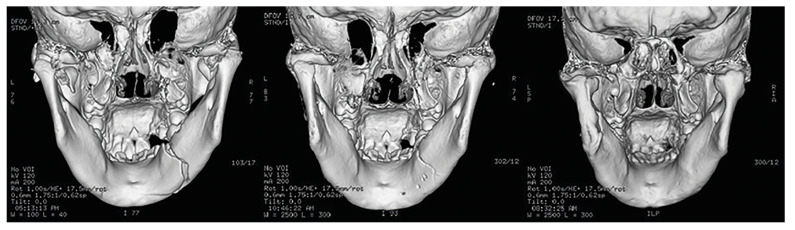
CT images of a case with severe dislocation of condylar fractures that underwent treatment of removing fracture fragments (left: before treatment; middle: 1 week after treatment; right: 6 months after treatment).

**Figure 3:**
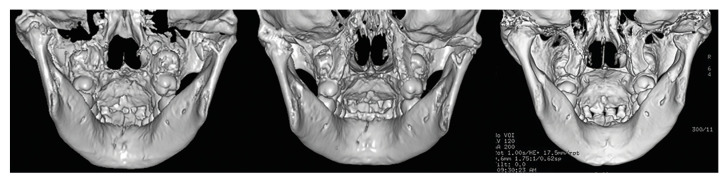
CT images of a case with comminution of condylar fractures that underwent surgery to remove fracture fragments (left: before surgery; middle: 1 week after surgery; right: 6 months after surgery).

One-week postoperative radiographic images showed that the fracture fragments were removed in these patients (Fig. 2, Fig. 3). Postoperative radiographic follow-up revealed a gradual return to the normal morphology in the condyles (Fig. 2, Fig. 3).

During the entire follow-up period, no abnormal reactions, rarefaction or sequestration in the bone were observed in the radiographic images of all cases. Spiral multi-slice CT images 3 and 6 months after the surgery showed complete bone healing on the fractured side, signs of ankyloses of the TMJ, and apparent mandibular or facial asymmetries were not seen. Moreover, a surprising phenomenon was observed: a partial or complete condyle was regenerated where the fragments were removed—the newly regenerated condyles presented spherical shapes with joint surfaces.

Summaries of the radiographic results are described in Table 2. Radiographic evaluation revealed no further loss of MRH on both left and right sides throughout the follow-up period (*p* = 0.456 and *p* = 0.484, respectively). An optimal condylar head remodelling was achieved in most cases. It turned out that the APD and MLD of the condylar head at long-term follow-up differ significantly from the values measured postoperatively on bilateral condyles (*p* = 0.029 and *p* = 0.012, respectively, for the left side; *p* = 0.039, and *p* = 0.043, respectively, for the right side).

Measurements of the differences between bilateral condyles in the ramus height and anteroposterior and mediolateral aspects were taken to assess the symmetry (Table 3). At the last follow-up, the APD of the condylar head was normal or showed slight asymmetry, and only one case of moderate asymmetry was observed. Four of eight postoperative asymmetry cases showed significant improvement in ramus height, while only one showed moderate asymmetry. The MLD of the condylar head in four cases, which showed moderate asymmetry postoperatively, has significantly improved.

## Discussion

The treatment of paediatric condylar fractures is one of the most contradictory themes explored by surgeons. Several elements, such as age, mixed dentition, the type and sites of fracture, and the presence of a condyle growth centre, must be considered when formulating a treatment plan for condylar fractures in children (14). Decisions on treating paediatric condylar fractures remain debated in oral and maxillofacial surgery practice. Controversies in surgical management arise from its pronounced disadvantages, such as complicated surgical procedures, possible injury to the facial nerve and vessels, aesthetically unaccepTable scars and postoperative infection. Most surgeons prefer conservative treatments, MMF accompanied by physical therapy, for instance, as they provide satisfactory results in most cases of paediatric condylar fractures. However, complications after condylar fractures, such as facial asymmetry and TMJ ankylosis, have provoked controversy. Besides, MMF is difficult to use in children, considering the inadequate number of primary teeth and incomplete eruption of permanent teeth (15).

This study is novel in confirming that removing fracture fragments is a promising approach for treating intracapsular condylar fractures in paediatric patients. This is the first report of this new treatment method globally. Although this method is not widely applied, the observed possibilities are encouraging. No occlusal disturbance, limited mouth opening, or mandibular growth disturbance was observed after surgical intervention. Furthermore, even surprisingly, regeneration of a new condylar head was observed. The radiographs demonstrated a significantly better outcome. The measurements of condyle dimension showed that the condylar head regained mediolateral and anteroposterior thickness in all cases at the last follow-up. Only one case with unilateral comminuted condylar fracture showed moderate asymmetry of the condylar head in anteroposterior aspects after long-term follow-up, which might be interpreted as severe damage to the condylar head and loss of the articular cartilage on the fractured side. At the same time, the height of the mandibular ramus remained unchanged at the last follow-up compared with the values measured postoperatively. Following the removal of the fragments, there may be an inadequate vertical height restoration, which possibly causes occlusion disturbance or overloading of the contralateral condyle (16). AccepTable results were obtained in our study, and four of seven slight asymmetry cases returned to normal condition.

The remaining three cases remained approximately the same due to a relatively short follow-up period. Neff *et al*. considered the fracture location on the condyle a determining factor in the clinical and radiographical outcomes, which coincided with our study (17). A less favourable prognosis appeared in one case with a fracture line running through the lateral condyle, displaying moderate asymmetry in ramus height postoperatively and at long-term follow-up, as the support of the lateral condyle part could not maintain the vertical dimensions. Based on the satisfactory outcomes of the present study, we can affirm that the removal of the fragments is a reliable and practical approach to restoring mandible function and promoting condyle regeneration in managing intracapsular condylar fractures in children.

Absolute indications for surgical management of paediatric condylar fracture are uncertain. We suggest that the indications of this new method reported in this study should include intracapsular condylar fractures in children with comminution of the condylar head or severe dislocation of the condylar fracture fragments out of the fossa, which can cause the interference of mandibular movement and persistent joint pain during mouth opening.

The rationality of applying this new method can be described as follows. The fractured condyle is surrounded by blood clots and fibrous callus tissues, which may induce TMJ fibrosis or ossification (18). Once the TMJ mobility is decreased, the risk of fibrous and bony TMJ ankylosis will increase. The blood clot, fibrous callus tissue and fracture fragments can be thoroughly removed by surgical operation on a complete debridement basis, thereby preventing the development of postoperative TMJ ankylosis.

The fracture fragments between the fossa and the mandibular ramus may lead to restricted mouth opening and pain during mandible movements. Non-compliance and low pain threshold of children may cause failure to carry out conservative treatment. These patients usually refuse to cooperate with postoperative mouth-opening exercises both actively and passively, which will result in further restriction of mouth-opening and even TMJ ankylosis. Removing the fractured fragments can eliminate resistance to mandibular movement and alleviate pain, thereby helping to optimise clinical outcomes. Moreover, residual fragments may affect the healing morphology of the fractured condyle, impacting the joint function. Yamashita *et al*. elucidated that though most of the crushed small fracture fragments had fused in cases of comminuted condylar fracture after conservative treatment, the rest of the fragments remaining individually detached would subsequently bring about dysfunction and pain during chewing (19).

Intracapsular condylar fractures, especially comminution of the condylar head and severe dislocation of fracture fragments cause bone and articular peripheral soft tissue injury, including associated attachment damage, disc destruction or displacement. The articular disc displacement, tear or perforation and absence of an intact disc associated with condylar fractures are significant determinants in the development of TMJ ankylosis (20). The location of the disc at the fracture site is primarily important; the disc typically serves as a barrier to prevent the fusion of the distal fragments with the joint fossa, and ankylosis generally occurs if this relationship is not maintained (21). The fractured condyle, with its surface presented rough and sharp, will aggravate injury to the disc, causing the disc perforation or fibrous adhesion, possibly resulting in TMJ ankylosis.

For these reasons, we hypothesise that inadequate management of the injured disc may be partly responsible for the poor prognosis of some cases of conservatively treated paediatric condylar fractures. Surgical intervention can pursue anatomical restoration and repair of the injured disc, while conservative management is insufficient. The healing of condylar fractures is closely related to the injury degree of the disc and its associated attachment in a period of growth. If the disc could be retained, repaired and repositioned, related to removing the fracture fragments, which hinder the mandibular movement, then the normal disc-condyle relationship would be reconstructed to the most extent to accelerate the function restoration and further growth in children.

The condyle is a centre of growth and development for the mandible with marked potential for remodelling and regeneration in children under twelve (22). Experimental studies and clinical observations have demonstrated that the condyle suffering trauma has a high capacity for regeneration and remodelling (23,24). Fractures heal more rapidly with fewer complications due to the high osteogenic potential of children. This finding explains the satisfactory morphology and function of the fractured condyle after surgical intervention. Moreover, it is indicated that the articular disc plays a pivotal role in condyle regeneration and mandibular growth (25). In the present study, regeneration of a new condylar head in a spherical shape with a joint surface was observed in cases with at least 3 months follow-up. The disc is predicted to possess the ability to induce condyle remodelling and regeneration, which is consistent with what was noted in animal experiments.

There were certain limitations in this preliminary study. Firstly, the number of cases was relatively small, insufficient to conclude complications such as TMJ ankylosis. Many classic studies focus on conservatively managing condylar fractures in children, involving sufficient cases and achieving reliable conclusions (8,9). Compared with these studies, the number of patients in our study was relatively small. However, our study dealt only with selected intracapsular condylar fractures with comminution or severe dislocation and restricted mouth opening. More data are needed to draw firmer conclusions in our following study. Next, more cases will be included in our study. Secondly, the mean follow-up period was 13.6 months and, therefore, relatively short. The bone growth capacity in children is quite large, and the participants should be followed up longer. More cases and long-term follow-ups are required to ascertain if this new approach should be extensively applied in clinical practice.

## Conclusions

The proposed approach offers a new perspective on managing intracapsular condylar fractures in paediatric patients. Analysis of the clinical and radiographic results of the present study indicated that removing the fracture fragments effectively delivered a satisfactory clinical effect and permitted enduring condyle remodelling and regeneration in a short period.

## Figures and Tables

**Table 1:** Pre- and postoperative summary of patients in this study.

No.	Age	Gender	Follow-up (months)	Pre.MO (finger)	Post.MO (finger)	Condylar fracture condition	Condyle condition in follow-up
1*	6	F	45	2	3	Severe dislocation, no comminution	PRC
2*	7	M	24	1	3	Comminution	CRC
3*	10	M	3	2	3.5	Severe dislocation, no comminution	PRC
4^#^	12	M	3	2	3.5	Severe dislocation, no comminution	CRC
5^#^	12	F	8	0.5	3	Severe dislocation, no comminution	PRC
6**	5	M	24	0.5	3	Comminution	CRC
7**	8	M	6	1.5	3	Severe dislocation, no comminution	PRC
8**	11	M	3	1	3	Severe dislocation, no comminution	PRC
9**	12	M	7	0.5	3.5	Severe dislocation, no comminution	CRC

* Unilateral fractures; ^# ^Bilateral fractures with one side treated with removal of the fragments; ** Bilateral fractures with removal of the fragments; M: male; F: female; Pre.MO: preoperative mouth opening; Post.MO: postoperative mouth opening; CRC: Complete regenerated condyle; PRC: Partial regenerated condyle.

**Table 2:** Summaries of the radiographical results.

	Left side		*p*	Right side		*p*
	Postoperatively	Long-term follow-up		Postoperatively	Long-term follow-up	
MRH (mm)	51.23±4.62	51.37±5.49	0.456	53.57±4.99	53.51±5.43	0.484
MLD (mm)	14.47±2.66	17.96±2.76	0.029	15.01±4.43	18.21±2.99	0.039
APD (mm)	8.38±1.18	11.91±2.31	0.012	8.83±1.79	10.64±1.88	0.043

Significant at *p*< 0.05.

**Table 3:** Comparison of MRH and condyle dimensions between the left and right sides.

Differences between the left and right sides		Grade (n=9)			
		Normal	Slight	Moderate	Severe
MRH	Postoperatively	1	7	1	—
	Long-term follow-up	5	3	1	—
MLD	Postoperatively	4	1	4	—
	Long-term follow-up	6	3	—	—
APD	Postoperatively	8	1	—	—
	Long-term follow-up	6	2	1	—

Classification standard for the difference between the left and right sides: normal: ≤2mm; slight: 2-4mm; moderate: 4-8mm; severe: ≥8mm.
